# Supplementary material for: The genome sequence of the Small Cowberry Marble, Rhopobota ustomaculana (Curtis, 1831) (Lepidoptera: Tortricidae)
Source: Wellcome Open Res. 2026 Sep 24;11:749. [Version 1] doi: 10.12688/wellcomeopenres.27930.1 (PMC13736057; doi:10.12688/wellcomeopenres.27930.1)
Supplement: Peer review history

# Reviewer report: Reviewer response for version 1

**DOI:** 10.21956/wellcomeopenres.30730.r175737

**Published:** 2026-11-04

**Authors:** Natalia Florencio Martins

**Affiliations:** 1 Embrapa Agroindústria Tropical, Fortaleza, Brazil

The article reports the first chromosome-level genome assembly for *Rhopobota ustomaculana* (Small Cowberry Marble), a tortricid moth, and states that this is the only genome assembly currently available for the species and the RefSeq reference assembly. The assembly for a single adult male confirmed by morphological and DNA barconding assure the specimen gender. Aside to the nuclear chromossomes the work reports the mitochondrial genome assembled and curated. 

 The standard genome-assembly pipeline proved to be methodologicallly credible. It´s quality was supported by high quality and BUSCO completeness. 

 Nevertheless, the exact order and orientation of contigs in chromosome 14 are unknown in two regions, which could be mentioned and explored. One point that might be explored is the genome annotation as well as the remaining gaps in the sequence.

 In conclusion the genome sequence of the Small Cowberry Marble, *Rhopobota ustomaculana *present a very clear ensemble of results and an important contribution for Lepidoptera genomic data.

Are the datasets clearly presented in a usable and accessible format, and the assembly and annotation available in an appropriate subject-specific repository?

Yes

Are sufficient details of the sequencing and extraction, software used, and materials provided to allow replication by others?

Yes

Are the rationale for sequencing the genome and the species significance clearly described?

Yes

Are the protocols appropriate and is the work technically sound?

Yes

Reviewer Expertise:

Genomics and bioinformatics

I confirm that I have read this submission and believe that I have an appropriate level of expertise to confirm that it is of an acceptable scientific standard.

# Reviewer report: Reviewer response for version 1

**Published:** 2026-09-24

**Authors:** Automated Benchmarking

**Affiliations:** 1 Wellcome Sanger Institute, Hinxton, UK

As this article is a Genome Note, Automated Benchmarking has been used to determine whether the reported assembly passes four defined checks established by the Earth BioGenome Project: the raw and assembled data are publicly available, the Earth BioGenome Project three-part metric is met, the k-mer completeness exceeds 90%, and the BUSCO score passes the stated threshold. For Rhopobota ustomaculana (ilRhoUsto1), all four checks passed. Data available: pass. ENA study PRJEB82191, raw-read accessions ERR13946479 and ERR13947500, and assembly accessions GCA_964656095.1 and GCA_964656135.1 are public. Earth BioGenome Project three-part metric: pass. Haplotype 1 is reported as 6.C.Q64, meeting the 6.C.Q40 reference standard. k-mer completeness: pass. The combined haplotype assemblies have 99.25% k-mer completeness, exceeding the Earth BioGenome Project target of 90%. BUSCO: pass. Haplotype 1, which is proposed as the reference assembly for this species, has 98.2% complete BUSCOs (97.7% single-copy and 0.6% duplicated). BUSCO software version: 5.5.0; lineage dataset: lepidoptera_odb10. It meets the target of greater than 90% complete and single-copy BUSCOs. This decision uses values reported in the Genome Note and public accession records.

Reviewer Expertise:

NA

I confirm that I have read this submission and believe that I have an appropriate level of expertise to confirm that it is of an acceptable scientific standard.
